# CD57^high^ Neuroblastoma Cells Have Aggressive Attributes *Ex Situ* and an Undifferentiated Phenotype in Patients

**DOI:** 10.1371/journal.pone.0042025

**Published:** 2012-08-10

**Authors:** Anne-Marie Schlitter, Carmen Dorneburg, Thomas F. E. Barth, Joachim Wahl, Johannes H. Schulte, Silke Brüderlein, Klaus-Michael Debatin, Christian Beltinger

**Affiliations:** 1 Department of Pediatrics and Adolescent Medicine, University Medical Center Ulm, Ulm, Germany; 2 Institute of Pathology, University Medical Center Ulm, Ulm, Germany; 3 Pediatric Hematology and Oncology, University Children's Hospital Essen, Essen, Germany; University of Chicago, United States of America

## Abstract

**Background:**

Neuroblastoma is thought to originate from neural crest-derived cells. CD57 defines migratory neural crest cells in normal development and is expressed in neuroblastoma.

**Methodology and Principal Findings:**

We investigated the role of CD57 expression in neuroblastoma cells *ex situ* and *in situ*. Compared to CD57^low^ U-NB1 neuroblastoma cells, CD57^high^ cells developed tumors with decreased latency after orthotopic transplantation into adrenal glands of mice. In addition, CD57^high^ U-NB1 and SK-N-BE(2)-C neuroblastoma cells were also more clonogenic, induced more spheres and were less lineage-restricted. CD57^high^ cells attached better to endothelial cells and showed enhanced invasiveness. While invasion of U-NB1 cells was inhibited by blocking antibodies against CD57, neither invasion of SK-N-BE(2)-C cells nor adhesion of U-NB1 and SK-N-BE(2)-C cells was attenuated. After tail vein injection only CD57^high^ cells generated liver metastases, while overall metastatic rate was not increased as compared to CD57^low^ cells. In stroma-poor neuroblastoma of patients CD57^high^ cells were associated with undifferentiated tumor cells across all stages and tended to be more frequent after chemotherapy.

**Conclusion:**

Strong expression of CD57 correlates with aggressive attributes of U-NB1 and SK-N-BE(2)-C neuroblastoma cells and is linked with undifferentiated neuroblastoma cells in patients.

## Introduction

High-risk neuroblastoma (NB) is an aggressive tumor of childhood whose prognosis has improved little for decades despite intensive multimodal therapy [1]. NB is an embryonic tumor thought to arise from immature derivatives of neural crest stem cells (NCSC) [2], [3], [4]. CD57, detected by the HNK-1 antibody [5], is a carbohydrate epitope expressed on adhesion molecules of NCSC and is associated with migration of these cells and their invasion and colonisation of tissues during development [6], [7]. Thus, in NCSC, expression of CD57 is linked to a cellular behavior that also characterizes aggressive neuroblastoma. In line with this notion, strong expression of CD57 has been associated with histologically more immature NB cells [8]. However, experimental evidence investigating the role of strong CD57 expression in the aggressiveness of NB is lacking. We therefore set out to address this question using the short term NB culture U-NB1 and the NB cell line SK-N-BE(2)-C. We show that within these NB those cells selected for strong expression of CD57 have more aggressive attributes *ex situ*. In patients with stroma-poor NB strong expression of CD57 is associated with undifferentiated cells across disease stages and with residual tumor cells after chemotherapy.

## Results

### U-NB1 cells strongly expressing CD57 show enhanced clonogenicity and induction of spheres

**Figure 1:**
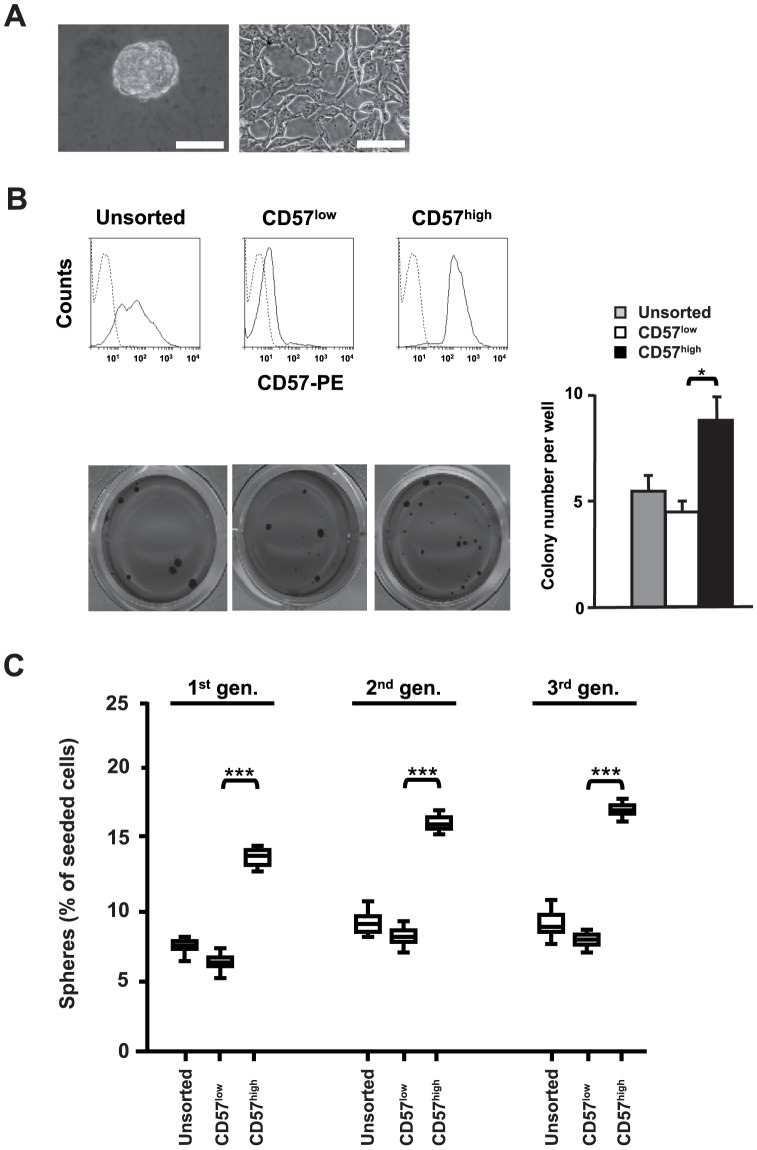
Strong expression of CD57 in U-NB1 neuroblastoma cells promotes clonogenicity and induction of spheres. (**A**) **Morphology of U-NB1 cells growing in serum-free and serum-replete medium.** Appearance of spheres derived from single cells plated in non-adherent plates and grown in serum-free medium containing EGF and bFGF (left panel). Morphology of cells when cultured on collagen-coated plastic in serum-supplemented medium (right panel). Bars correspond to 30 µm. (**B**) **Enhanced clonogenicity of U-NB1 cells strongly expressing CD57.** U-NB1 cells were separated by flow cytometry into a CD57^high^ fraction (cells above the 80^th^ percentile of CD57 expression) and a CD57^low^ fraction (cells below the 20^th^ percentile). CD57 expression of unsorted and sorted cells is shown in histograms (upper panel). Solid lines correspond to CD57, dashed lines to isotype controls. For soft agar clonogenicity assays fractionated U-NB1 cells were seeded at 1000 cells per well into 24-well plates. 28 days after plating colonies were visualised by MTT staining. The numbers of colonies per well are depicted in the graph. The asterisk denotes p<0.05, using Student's t-test. Experiments were repeated three times, with similar results. (**C**) **Enhanced induction of spheres by U-NB1 cells strongly expressing CD57.** U-NB1 cells were separated by flow cytometry into CD57^high^ and CD57^low^ fractions. Fractionated as well as unsorted cells were plated at very low density in serum-free medium. The percentage of “first generation” spheres growing from single cells was determined. After dissociation of spheres this procedure was repeated twice (“second” and “third generation” spheres, respectively). Results are shown as box plots (lower panel). Asterisks denote p<0.001, using Student's t-test. Experiments were performed twice, with similar results.

Clonogenicity correlates with the aggressiveness of tumors. The capacity of neuroectodermal tumor cells to initiate spheres under serum–free conditions is associated with their capacity to initiate tumors [9], [10]. We therefore determined, whether the expression of CD57 in NB cells is associated with clonogenicity and induction of spheres. We first investigated NB cells without amplification of *MYCN* or deletion of 1p (U-NB1; Fig. 1A). These cells were established and maintained as spheres in serum-free medium, a procedure known to preserve the characteristics of the parental tumor [11]. 56% of U-NB1 cells expressed CD57, as determined by flow cytometry (Fig. 1B, upper left panel). CD57 expression was distributed in a bimodal fashion among U-NB1 cells, suggesting two subpopulations in regard to CD57 expression. This may be due to the fact that the U-NB1 culture, which has been propagated as spheres and with a low passage number, mirrors the heterogeneity of tumor cells found in patients' tumors. U-NB1 cells were fractionated by FACS into a CD57^high^ fraction (i.e., cells above the 80^th^ percentile of CD57 expression) and a CD57^low^ fraction (cells below the 20^th^ percentile) (Fig. 1B, upper panel). While the clonogenicity of U-NB1 cells and its fractions was very low, there was a significant increase in clonogenicity of CD57^high^ compared to CD57^low^ cells (Fig. 1B, lower panels). CD57^high^ cells also induced spheres at a 2.8–fold higher frequency than did CD57^low^ cells (Fig. 1C). Upon dissociation of CD57^high^-derived spheres, cells fractionated again or left unfractionated induced new spheres with increased frequency upon recloning (p<0.001), suggesting that cells growing within spheres become adept at inducing new spheres. More important, CD57^high^ cells were always superior to CD57^low^ cells in inducing spheres.

Taken together, high expression of CD57 marks a population within U-NB1 cells with increased clonogenicity and an enhanced and sustained disposition to induce spheres. This suggests increased aggressiveness of CD57^high^ U-NB1 cells.

### Clonogenicity and sphere induction of SK-N-BE(2)-C NB cells correspond to expression of CD57

**Figure 2:**
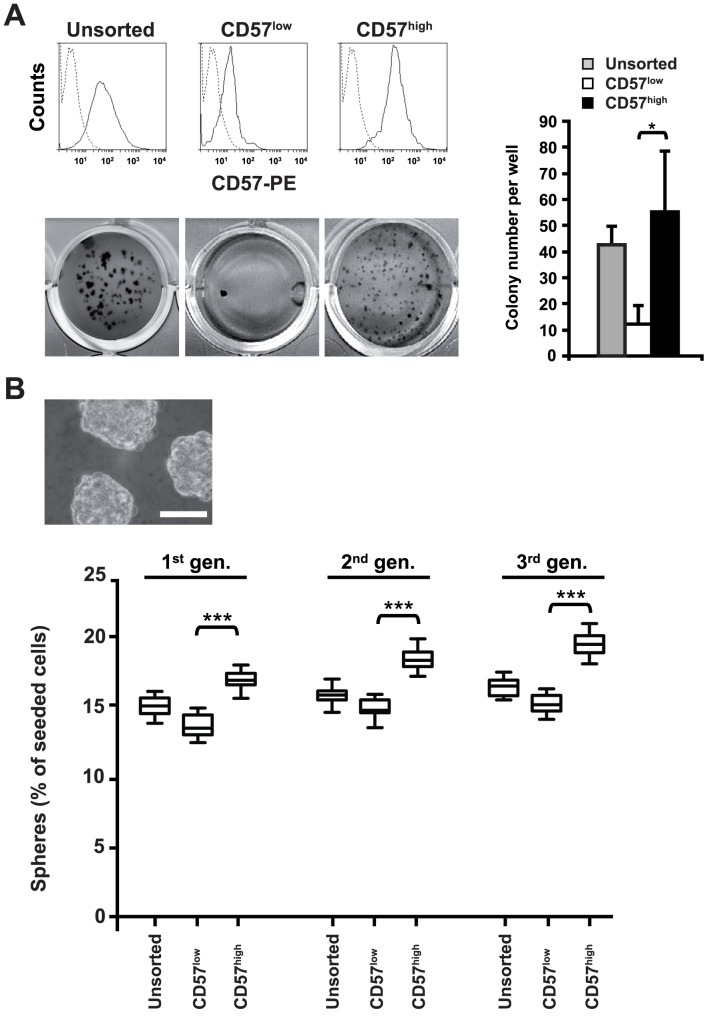
Expression of CD57 determines clonogenicity and correlates with induction of spheres **of SK-N-BE(2)-C neuroblastoma cells.** (**A**) **SK-N-BE(2)-C cells with high expression of CD57 are clonogenic.** SK-N-BE(2)-C cells were separated by flow cytometry into CD57^high^ and CD57^low^ fractions. For soft agar clonogenicity assays fractionated SK-N-BE(2)-C cells were seeded at 2000 cells per well into 24-well plates. 28 days after plating, colonies were visualised by MTT staining. The numbers of colonies per well are depicted in the graph. The asterisk denotes p<0.05, using Student's t-test. Experiments were repeated three times, with similar results. (**B**) **CD57 expression of SK-N-BE(2)-C cells correlates with induction of spheres.** The propensity of single SK-N-BE(2)-C cells to generate spheres when plated at very low density in serum-free medium was compared between unsorted SK-N-BE(2)-C cells (upper panel, bar equals 30 µm), CD57^high^ cells and CD57^low^ cells. Results are calculated as the percentage of spheres generated from the cells seeded and are shown as box plots (lower panel). Asterisks denote p<0.001, using Student's t-test. Experiments were performed twice, with similar results.

We extended our study by investigating a *MYCN*-amplified NB cell line with deletion of 1p, SK-N-BE(2)-C. Flow-cytometric analysis showed that 70% of SK-N-BE(2)-C cells expressed CD57 in Gaussian distribution (Fig. 2A, upper left panel). SK-N-BE(2)-C cells were sorted into CD57^low^ and CD57^high^ fractions (Fig. 2A, upper middle and right panels). Clonogenicity of SK-N-BE(2)-C cells clearly segregated with expression of CD57, as clonogenic growth was confined to the CD57^high^ fraction, with very few colonies growing in the CD57^low^ fraction (Fig. 2A, lower panels).

Expression of CD57 also correlated with the capacity of SK-N-BE(2)-C cells to initiate first, second and third generation spheres in serum-free medium (Fig. 2 B).

Compared to U-NB1 cells (Fig. 1) clonogenicity and, less so, sphere forming capacity of SK-N-BE(2)-C cells were higher. As in U-NB1 cells, strong expression of CD57 endows SK-N-BE(2)-C cells with increased clonogenicity and enhanced sphere induction, *in vitro* attributes of an aggressive cellular phenotype.

### Increased frequency of peripherin^−^ GFAP^−^ cells in CD57^high^ U-NB1 and SK-N-BE(2)-C cells

**Figure 3:**
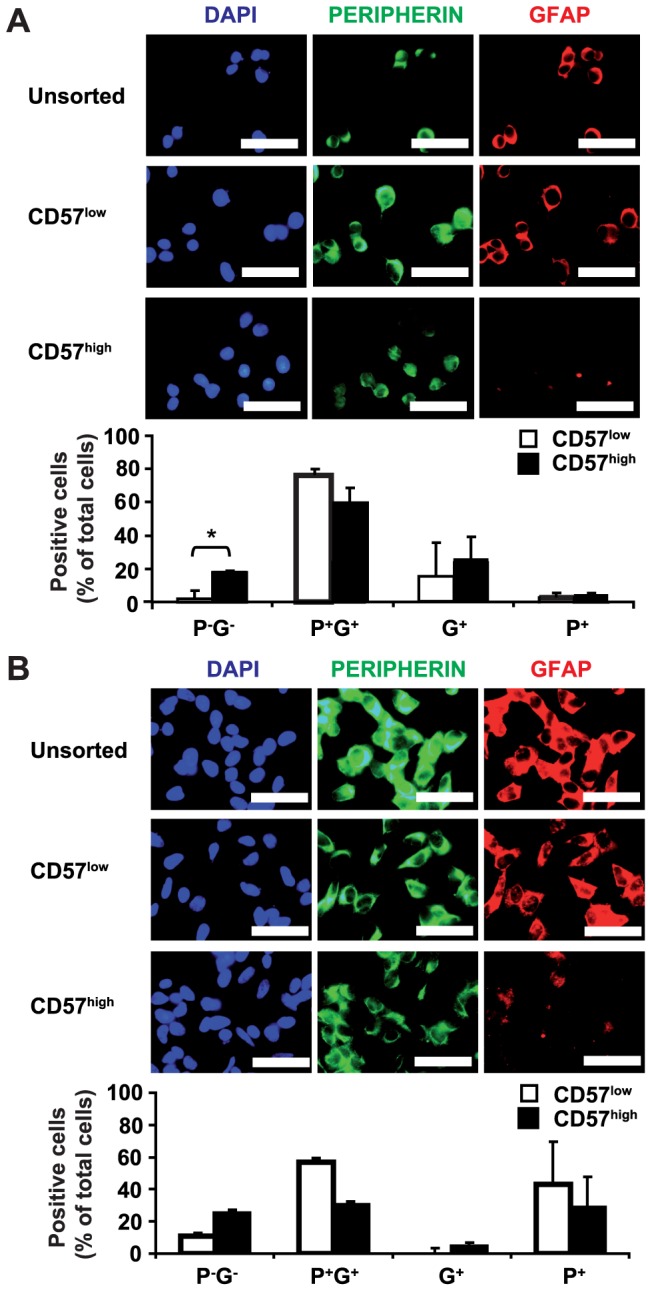
Increased frequency of peripherin^−^ GFAP^−^ cells in the CD57^high^ fraction of U-NB1 and SK-N-BE(2)-C cells. CD57^low^ and CD57^high^ U-NB1 cells (**A**) and SK-N-BE(2) cells (B) were isolated by FACS sorting. Cells were plated on cover slips and simultaneously stained for peripherin (green) and GFAP (red). Nuclei were visualized with DAPI (blue). Scale bars correspond to 50 µm. The results of the stainings were quantified in the graphs (P, peripherin; G, GFAP). The asterisk denotes p<0.05 using Student's t-test. Three independent experiments were performed, with similar results.

Less lineage-restricted and thus less mature tumor cells are usually more aggressive. We therefore analyzed, whether expression of CD57 is associated with a less lineage-restricted phenotype of U-NB1 and SK-N-BE(2)-C cells. U-NB1 cells have the morphology of so-called I-type NB cells (Fig. 1A, right panel), i.e., less lineage-restricted NB cells known to coexpress markers for both neuronal and non-neuronal lineages of the neural crest [12]. We therefore determined expression of peripherin, a neuronal marker, and glial fibrillary acidic protein (GFAP), a marker also expressed in glial cells, in U-NB1. Indeed, the majority of U-NB1 coexpressed peripherin and GFAP (Fig. 3A). We next investigated how expression of CD57 in U-NB1 cells correlates with the expression of peripherin and GFAP. Significantly more CD57^high^ cells were negative for both peripherin and GFAP compared to CD57^low^ cells (Fig. 3A). No significant differences were found in the percentage of cells double- or single-positive for peripherin and GFAP.

We showed that many SK-N-BE(2)-C cells, known I-type NB cells, also coexpress peripherin and GFAP. CD57^high^ SK-N-BE(2)-C cells tended to harbor more peripherin^−^ GFAP**^−^** cells, although this trend was not statistically significant (Fig. 3B).

Taken together, the CD57^high^ fraction of U-NB1 contains significantly more double-negative cells than the CD57^low^ fraction, while SK-N-BE(2)-C cells show a trend in this direction. Assuming that double-negative cells are less lineage-restricted and thus less mature than double-positive cells this suggests increased aggressive potential of CD57^high^ cells.

### CD57^high^ but not CD57^low^ U-NB1 and SK-N-BE(2)-C cells change lineage attributes upon forced differentiation *in vitro*

**Figure 4:**
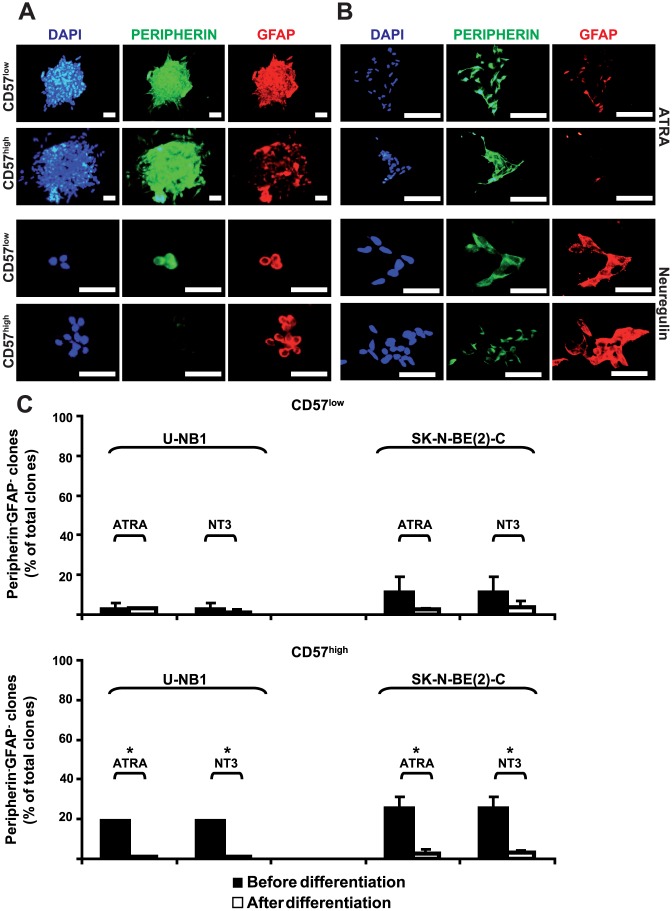
CD57^high^ but not CD57^low^ U-NB1 and SK-N-BE(2)-C cells are responsive to inducers of neuronal or glial lineage attributes. CD57^low^ and CD57^high^ U-NB1 cells (**A**) or CD57^low^ and CD57^high^ SK-N-BE(2)-C cells (B) were isolated by FACS. Cell fractions were plated in clonal density onto collagen-coated cover slips. Cells were treated over a ten day period with ATRA or with NRG1–ß1 for 14 days in medium supplemented with 2% FBS, followed by double–immunofluorescence staining for peripherin (green) and GFAP (red). Nuclei were stained with DAPI (blue). (**C**) Frequency of peripherin/GFAP double-negative clones upon forced differentiation as in (A) and (B). The asterisks denote p<0.05 using Student's t-test. Scale bars correspond to 50 µm. All experiments were repeated at least three times, with similar results.

Next we investigated the response of the fractions to forced *in vitro* differentiation towards attributes of peripheral sympathetic neurons and glial Schwann cells. Clones of CD57^low^ and CD57^high^ U-NB1 cells (Fig. 4A) and SK-N-BE(2)-C cells (Fig. 4B) were exposed to all-trans-retinoic acid (ATRA) and neuregulin for neural and glial differentiation, respectively. CD57^low^ cells were unresponsive to these agents, as expression of peripherin and neuregulin did not change. In contrast, CD57^high^ cells responded to the neuralizing ATRA by maintaining peripherin expression and decreasing expression of GFAP. The response of CD57^high^ cells to neuregulin was also pronounced, as expression of peripherin was markedly attenuated while GFAP expression was maintained. Thus, CD57^high^ U-NB1 and SK-N-BE(2)-C cells have an increased capacity to change lineage attributes upon forced neuronal and glial differentiation *in vitro*, which may suggest a less mature state of CD57^high^ cells.

Also of note, the number of double-negative clones significantly decreased upon forced differentiation of CD57^high^ U-NB1 and SK-N-BE(2)-C clones and tended to decease in CD57^low^ SK-N-BE(2)-C clones (Fig. 4 C). This is consistent with double-negative cells being more responsive to differentiation cues and thus supports the notion that double-negative cells are less lineage-restricted and hence less mature.

### CD57^high^ U-NB1 cells initiate orthotopic tumors with shorter latency than CD57^low^ cells

**Figure 5:**
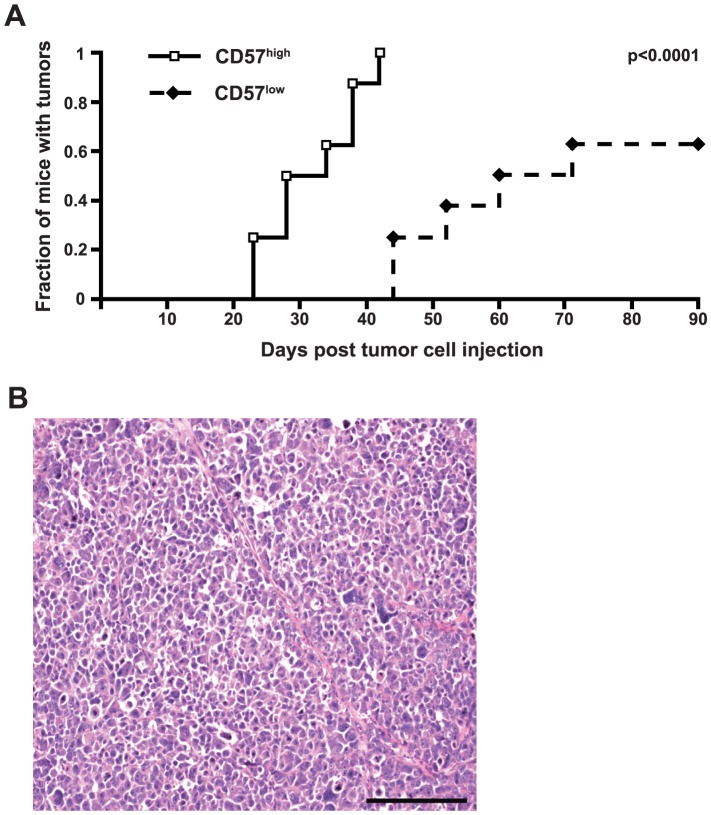
Enhanced formation of stroma-poor neuroblastoma by CD57^high^ U-NB1 cells. (**A**) **Enhanced tumor formation of CD57^high^ U-NB1 cells.** CD57^high^ and CD57^low^ tumor cells were isolated by FACS from one primary orthotopic tumor. 1×10^4^ cells were surgically transplanted into the left adrenal glands of immunodeficient mice (8 animals per group). The development of tumors was monitored by palpation and verified by necropsy when mice had to be killed because of excessively large tumors or when the experiment was terminated at 90 days. The log rank test was used for statistical analysis. The experiment was repeated twice, with similar results. (**B**) **Orthotopic U-NB1 tumors are stroma-poor.** Tumors were formalin-fixed, paraffin-embedded and HE-stained. Scale bar equals 200 µm.

Tumor latency and tumorigenicity were investigated in an orthotopic model by transplanting FACS-separated CD57^high^ and CD57^low^ U-NB1 cells from one primary orthotopic tumor into the adrenal glands of mice (n = 8 for each group). CD57^high^ cells showed decreased latency of tumor formation compared to CD57^low^ cells (Fig. 5A, p<0.001), demonstrating increased aggressiveness of CD57^high^ U-NB1 cells. The incidence of tumor formation by CD57^high^ cells at the end of the experiment was not significantly different from CD57^low^ cells (8/8 vs. 5/8 mice, respectively, p = 0.24407 using Fisher's exact test). The tumors that developed consisted of densely packed small round blue cells typical of stroma-poor neuroblastic neuroblastoma (Fig. 5B).

### CD57^high^ U-NB1 and SK-N-BE(2)-C cells have a higher propensity to invade and to adhere than CD57^low^ cells

**Figure 6:**
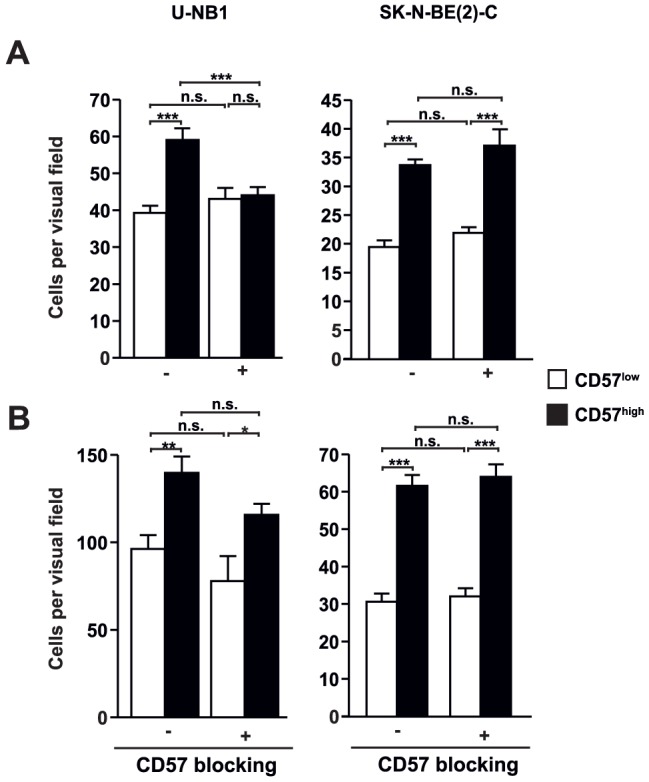
CD57^high^ NB cells are more invasive and adhesive than CD57^low^ cells. (**A**) **Increased invasion and migration of CD57^high^ NB cells in matrigel.** CD57^low^ and CD57^high^ fractions of U-NB1 and SK-N-BE(2)-C cells were labeled with DiI , incubated with 30 µg/ml of active (+) or heat-inactivated (−) blocking anti-CD57 antibody and added to matrigel-coated transwell chambers. The upper chamber was serum-free and the lower chamber contained serum. 24 h post seeding cells that had invaded into and migrated within in the matrigel were fixed. The number of invasive cells is expressed as cells per visual field (200× magnification). * p<0.05, ** p<0.01, *** p<0.001 and n.s. = not significant using Student's t-test. Results are shown as means and standard deviations of three independent experiments using duplicates. (**B**) **Enhanced adhesion of CD57^high^ NB cells to HUVEC.** U-NB1 and SK-N-BE(2)-C cells were FACS-sorted into fractions with low and high expression levels of CD57. After sorting, U-NB1 and SK-N-BE(2)-C cells were labeled with DiI, incubated with 30 µg/ml of active (+) or heat-inactivated (−) blocking anti-CD57 antibody and seeded on a confluent monolayer of HUVECs. One hour post seeding cells were fixed and the number of adhering tumor cells per visual field (100× magnification) was determined. Results are shown as means and standard deviations of three independent experiments using duplicates. * p<0.05, ** p<0.01, *** p<0.001 and n.s. = not significant using Student's t-test. Results are shown as means and standard deviations of three independent experiments using duplicates.

As CD57 expression defines migrating and colonizing neural crest cells during development we reasoned that CD57 may also be involved in invasion and adherence of neuroblastoma cells since these are derived from neural crest cells. To this end, we determined invasion of CD57^high^ and CD57^low^ U-NB1 and SK-N-BE(2)-C cells into matrigel and their adhesion to human umbilical vein endothelial cells (HUVEC). CD57^high^ U-NB1 and SK-N-BE(2)-C cells showed an increased capacity to attach to and foray into extracellular matrix (Fig. 6A) and to adhere to HUVEC (Fig. 6B). To determine, whether strong expression of CD57 caused this behavior, invasion and adherence assays were also performed in the presence of blocking anti-CD57 antibodies. Proper function of the blocking antibody was confirmed by proving that it inhibited migration of a glioma cell line overexpressing CD57 but not of the parental cell line devoid of CD57 (Suppl. Fig. S1 A, B). Enhanced matrix invasion of CD57^high^ U-NB1 cells but not of CD57^high^ SK-N-BE(2)-C cells was inhibited by blocking CD57 (Fig. 6 A). Increased endothelial adhesion of CD57^high^ cells was not attenuated by blocking CD57 in both U-NB1 and SK-N-BE(2)-C cells (Fig. 6 B). To exclude that the antibody used for sorting prevented the blocking antibody from binding to CD57 or that internalization of antibody-bound CD57 decreased CD57 on the cell surface we performed sequential staining of CD57 with the sorting antibody and with a different anti-CD57 antibody. The sorting antibody was rapidly internalized and new CD57 swiftly appeared on the cell surface hence ruling out confounding effects of the sorting antibody on the blocking experiments (Suppl. Fig. S1 C).

Taken together, strong expression of CD57 in U-NB1 and SK-N-BE(2)-C cells is associated with attributes of aggressive cellular behavior but does not generally cause them.

### Upon intravenous injection CD57^high^ U-NB1 cells are more proficient in forming liver metastases, as compared to CD57^low^ cells

**Figure 7:**
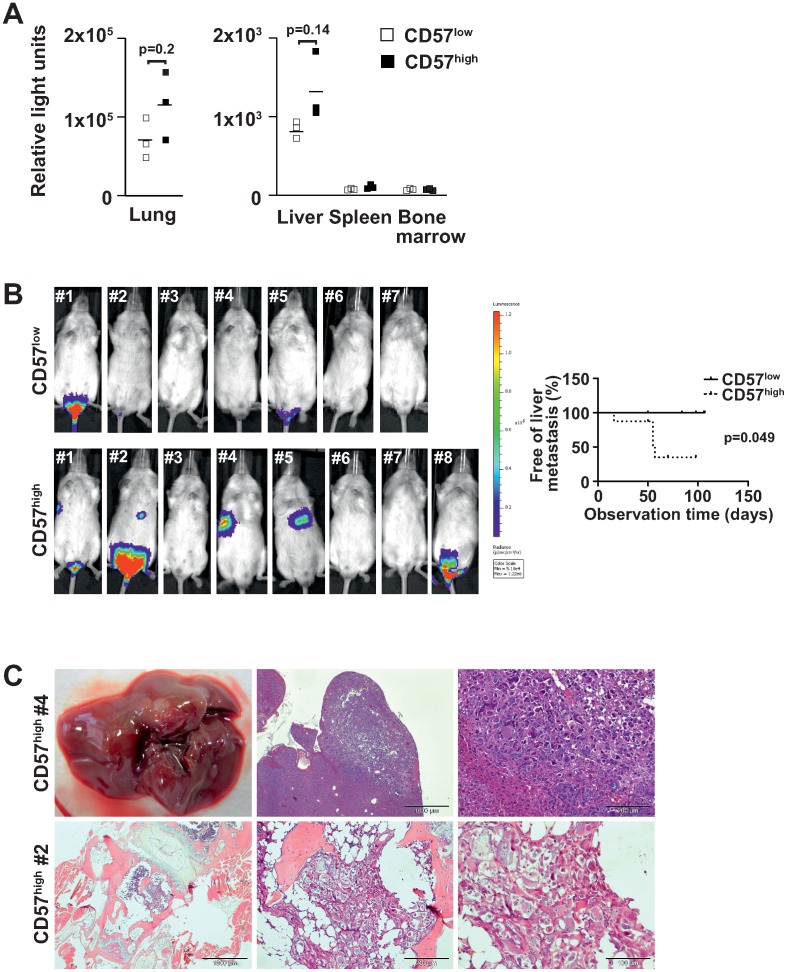
Only CD57^high^ cells generate liver metastases, while overall metastatic rate is not increased compared to CD57^low^ cells. (**A**) **CD57^high^ U-NB1 cells injected intravenously home mildly better to lung and liver than CD57^low^ cells.** U-NB1 cells from orthotopic tumors stably expressing the enhanced firefly luciferase gene were sorted into CD57^low^ and CD57^high^ fractions by flow cytometry. 5×10^5^ cells were injected into the tail vein of RAG−/− cgc−/− mice and the animals were sacrificed 30 min post injection. Organs were procured and lysed. Using a luciferase assay system luminescence was determined with a luminometer. Luminescence is expressed as relative light units. Because of different scales results are shown separately for lung and the other organs. Symbols represent the values of individual animals and bars the means. One representative of three independent experiments is shown. Student's t-test was used for statistical analysis. (**B**) **CD57^high^ cells generate liver metastases in addition to bone marrow metastases.** U-NB1 cells stably expressing firefly luciferase were sorted into CD57^low^ and CD57^high^ fractions by flow cytometry. 2×10^6^ cells were injected into the tail vein of NSG mice. Tumor development was assessed regularly beginning 21 days after tumor cell injection by luminescence imaging following luciferin injection. Mice are numbered for identification. The occurrence of liver metastases is depicted in the Kaplan-Meier plot, statistical analysis was performed using the log-rank test. (**C**) **Histology confirms bone marrow and liver metastases.** Mice were sacrificed 60–80 days post tumor cell injection and necropsy was performed. Organs were stained by H&E. Tail root of mouse CD57^high^ #2 and liver of mouse CD57^high^ #4 are shown.

Having shown *in vitro* that some cellular mechanisms important for metastasis, such as adherence and migration, are preferentially active in CD57^high^ NB cells, we investigated metastasis of CD57^high^ U-NB1 cells. Orthotopically transplanted U-NB1 cells did not spontaneously metastasize. We therefore employed an experimental metastasis model. CD57^low^ and CD57^high^ cells isolated from orthotopic U-NB1 tumors expressing luciferase were injected into the tail vein of mice. A trend towards increased homing to lung and liver of CD57^high^ cells as compared to CD57^low^ cells was observed 30 minutes after injection (Fig. 7A). Few tumor cells were detected in spleen and bone marrow.

To investigate, whether increased homing of the CD57^high^ U-NB1 cells translated into growth of metastases, CD57^high^ and CD57^low^ cells were isolated from orthotopic U-NB1 tumors expressing luciferase and injected into the tail vein of mice. *In vivo* bioluminescence imaging revealed that CD57^high^ cells initiated metastases in 5 out of 8 mice injected, whereas 3 metastases developed in the 7 mice injected with CD57^low^ cells (Fig. 7B, left panel; p = 0.62 using Fisher's exact test). 4 out of 8 mice with CD57^high^ cells but none of the mice with CD57^low^ cells developed liver metastases (Fig. 7B, left and right panel; p = 0.049). Most CD57^high^-initiated metastases were bigger than those of CD57^low^ cells (Fig. 7B). The luminescence signals corresponded to metastases detected by histology in liver and bone marrow (Fig. 7 C; and Suppl. Fig. S2). No microscopic metastases were found in organs negative for metastasis by bioluminescence imaging, confirming its specificity.

Taken together, these data show that strong expression of CD57 is associated with the ability of U-NB1 cells to form experimental liver metastases, without increasing overall metastatic rate.

### In patients strong expression of CD57 is associated with undifferentiated NB cells across disease stages and with post-chemotherapy residual tumor cells

**Figure 8:**
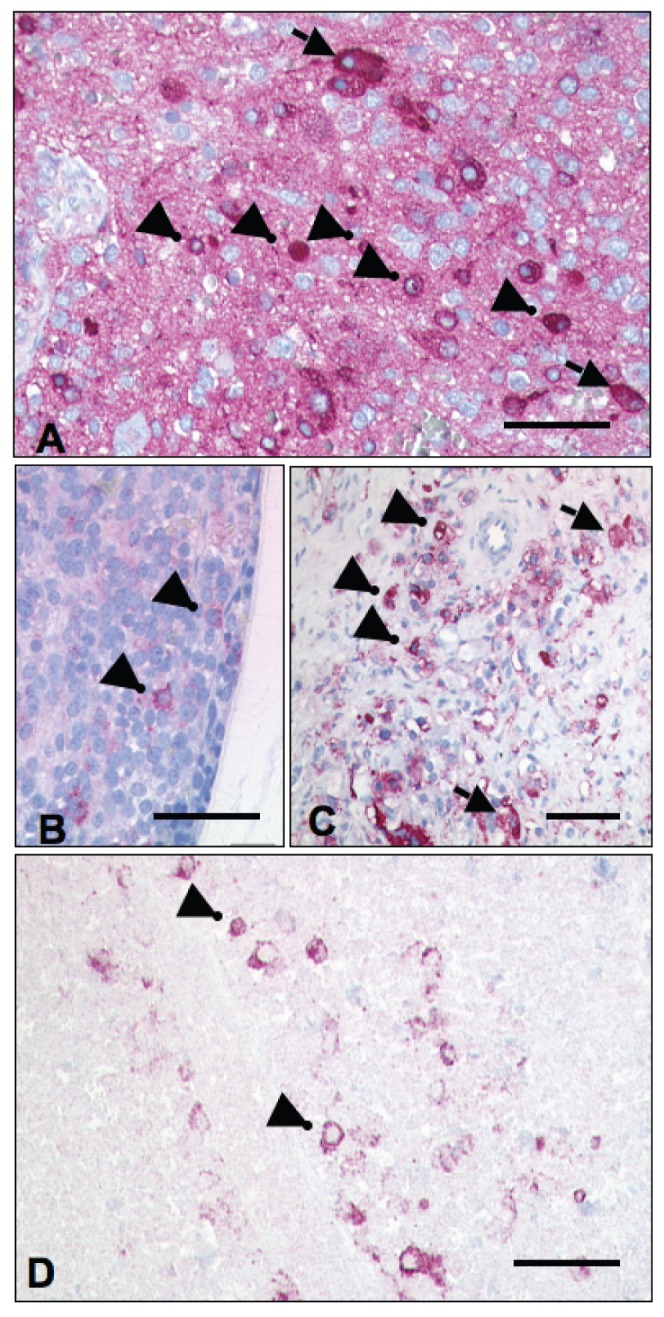
Strong expression of CD57 is linked with undifferentiated cells and exposition to chemotherapy in stroma-poor neuroblastoma of patients. (**A**) **More CD57^high^ cells in the undifferentiated compartment of NB.** Differentiating neuroblastoma (patient 3) with low background expression of CD57 in the neuropil. More undifferentiated cells (arrow heads) than larger, differentiating tumor cells (arrows) show high expression of CD57. Bars equal 70 µm for A–D. (**B and C**) **More CD57^high^ cells after chemotherapy.** Undifferentiated neuroblastoma bone marrow trephine (patient 12) prior to chemotherapy (B) with few CD57-positive cells (arrow heads). After chemotherapy (C) more tumor cells express CD57, with strong expression of CD57 predominantly found in undifferentiated cells (arrow heads) but only occasionally in larger, differentiating tumor cells (arrows). (**D**) **CD57^high^ cells remain in areas of chemotherapy-induced cell death.** Undifferentiated neuroblastoma after chemotherapy (patient 8). Residual CD57^high^ undifferentiated tumor cells (arrow heads) in the largely necrotic tumor.

To assess expression of CD57 in patient NB cells we investigated a panel of 16 NB that had been classified as Schwannian stroma-poor neuroblastoma according to the INPC classification (Table 1). Immunohistochemistry showed marked inter- and intratumoral heterogeneity of CD57 expression (Fig. 8 A and Table 1). Using the strong expression of CD57 in natural killer cells as a positive internal control for CD57^high^ cells we determined that strong expression of CD57 was significantly associated with cytologically undifferentiated NB cells (Fig. 8, Table 1, p<0.001 using the Mann-Whitney test). In line, 9 out 11 (82%; 95% confidence interval 48–98%) undifferentiated and poorly differentiated NB contained CD57^high^ cells, compared to 1 out 5 (20%; 95% CI 1–72%) differentiating NB. CD57^high^ cells at diagnosis were more often found in non-metastatic stage 1–3 NB (5 out of 6 (83%), 95% CI 36–100%) compared to metastatic stage 4 NB (4 out of 10 (40%), 95% CI 12–74%).

**Table 1:** CD57^high^ cells in Schwannian stroma-poor neuroblastoma of patients.

Patient	Localization	INPC classification	INSS stage	Undifferentiated CD57^high^ cells (% of all CD57^high^ cells)	CD57^high^ cells (% of tumor cells)	
					At diagnosis	After chemo
1	Adrenal	Poorly differentiated	1	90	60–70	-
2	Sympathetic trunk	Poorly differentiated	1	90	40	-
3	Adrenal	Differentiating	1	60	90	-
4	Adrenal	Poorly differentiated	2	70	50	-
5	Adrenal	Undifferentiated	3	100	80	50
6	Sympathetic trunk	Undifferentiated	3	100	<1	10
7	Adrenal	Differentiating	4	-	0	5
8	Adrenal	Undifferentiated	4	-	0	95
9	Bone	Undifferentiated	4	100	100	100
10	Paraaortic	Poorly differentiated	4	100	5	20
11	Adrenal	Differentiating	4	-	0	100
12	Bone, adrenal	Undifferentiated	4	100	<5	20–30
13	Lymph node	Differentiating	4	-	0	-
14	Abdomen	Differentiating	4	-	0	5
15	Liver	Poorly differentiated	4	80	50	-
16	Adrenal	Poorly differentiated	4	-	0	-

*INPC*, International Neuroblastoma Pathology Classification [21]; *INSS*, International Neuroblastoma Staging System [22]; *undifferentiated cells*, grade 3 NB cells according to Hughes classification (modified) [23]; *after chemo*, after chemotherapy as specified by the NB2004 Trial Protocol; *CD57^high^*, staining of CD57 at least as intense as in natural killer cells of the sample.

Paired pre- and post-chemotherapy NB samples showed a trend to increased frequency of CD57^high^ cells in post-chemotherapy residual tumors in 7 out of 8 informative tumor pairs (Fig. 8B, C and Table 1; two-tailed p = 0.11 using the paired-sample Wilcoxon signed rank test). This is also illustrated by Fig. 8D that shows residual CD57^high^ cells in a tumor area rendered largely necrotic by chemotherapy.

## Discussion

We show that CD57^high^ expressing neuroblastoma cells have aggressive attributes. Thus, we provide evidence that CD57^high^ cells from NB of different risk groups have enhanced sphere-inducing capacity and clonogenicity, a less mature phenotype, an increased response to lineage-altering agents, decreased tumor latency, enhanced invasive and adhesive properties and a propensity to generate experimental liver metastases. In stroma-poor NB of patients strong expression of CD57 was found preferentially in undifferentiated cells across disease stages at diagnosis and in residual tumor cells after chemotherapy.

### Increased clonogenicity and sphere-inducing capacity of CD57^high^ NB cells

Anchorage-independent growth in soft agar and, to a lesser degree, sphere forming capacity was more pronounced in SK-N-BE(2)-C cells compared to U-NB1 cells, maybe in part due to *MYCN* being amplified and 1p being lost in SK-N-BE(2)-C cells [13]. Importantly, both anchorage-independent growth as well as sphere forming capacity in low-serum was more pronounced in the CD57^high^ fraction of U-NB1 and SK-N-BE(2)-C. There was a weak and sustained increase of sphere-inducing capacity of CD57^low^ cells upon recloning. This may be explained by adaption of CD57^low^ cells to the conditions of sphere culture unrelated to CD57 expression. Primary and secondary spheres from CD57^high^ cells could be fractionated into CD57^high^ and CD57^low^ cells. This implies that CD57^high^ cells must have generated CD57^low^ cells during sphere growth. The ability of NB cells to grow as suspended spheroids in low-serum conditions may reflect AKT-mediated anoikis resistance [10], enhanced growth factor independence or other, yet to be identified mechanisms. Intercellular adhesion mediated by homophilic interaction of CD57 [14] may add to the inclination of CD57^high^ cells to form spheres.

Anchorage-independent clonogenic growth is a defining *in vitro* property of aggressive cancer cells and the ability of NB cells to induce spheres correlates with their aggressiveness [10]. Thus, the propensity of CD57^high^ U-NB1 and SK-N-BE(2)-C cells for clonogenic and spheroid growth reflects an increased aggressiveness of the CD57^high^ cell population.

### CD57^high^ U-NB1 and SK-N-BE(2)-C NB cells contain more immature cells

The CD57^high^ fraction of both U-NB1 and SK-N-BE(2)-C cells harbored more cells neither expressing peripherin nor GFAP, markers for sympathetic and glial cells, respectively. The number of these double-negative clones decreased upon forced differentiation, consistent with these clones being of a less lineage-restricted phenotype. Thus, the CD57^high^ fraction of U-NB1 and SK-N-BE(2)-C cells contains less mature cells. In addition, CD57^high^, but not CD57^low^ cells of both U-NB1 and SK-N-BE(2)-C could be forced to assume attributes of a predominant neuronal or glial phenotype. The increased capacity of these CD57^high^ NB cells to change lineage attributes provides additional support for the notion that this fraction contains less mature cells. Consistent with this notion, CD57 expression in SK-N-BE(2)-C cells has been shown to decrease upon differentiation to peripheral neuronal cells or glial cells [15]. The fact that CD57 marks neural crest stem cells, i.e., immature cells with increased capacity for differentiation (and sphere formation) lends further credibility to the association we observed in U-NB1 and SK-N-BE(2)-C cells between strong CD57 expression, less mature phenotype and increased capacity for changing lineage attributes (as well as enhanced sphere formation). The underlying molecular mechanisms for this association remain to be determined. As immaturity of cancer cells often correlates with enhanced aggressiveness, these findings may support the notion that U-NB1 and SK-N-BE(2)-C cells strongly expressing CD57 are more aggressive. Alternatively, the enhanced sensitivity of CD57^high^ NB cells to lineage cues may decrease these cells' aggressiveness should they encounter appropriate tumor-intrinsic or therapy-induced lineage cues.

### Enhanced invasion and decreased tumor latency of CD57^high^ NB cells

CD57^high^ U-NB1 and SK-N-BE(2)-C cells showed enhanced invasion into extracellular matrix *in vitro*. This may have contributed to the decreased tumor latency of CD57^high^ U-NB1 cells after orthotopic transplantation into adrenal glands of mice, the most common site of neuroblastoma. These data provide direct evidence of increased aggressiveness of CD57^high^ U-NB1 and SK-N-BE(2)-C neuroblastoma cells.

### Increased adhesive, colonizing and metastasizing propensity of CD57^high^ NB cells

Adherence of tumor cells to the endothelium of target organs, invasion of the subendothelial basal membrane and subsequent tumor cell growth are crucial late mechanisms in the metastatic cascade. We have shown that CD57^high^ U-NB1 and SK-N-BE(2)-C cells adhere better to endothelial cells and invade basal membrane matrix more extensively than CD57^low^ cells. While the matrigel trans-well chamber assay we employed cannot dissect the relative contributions of adhesion and migration to enhanced invasion of cells, the assay does show that invasion, the net result of adhesion and migration, is enhanced with CD57^high^ cells. After intravenous injection CD57^high^ U-NB1 cells were moderately more proficient in homing to and colonizing lung and liver.

Upon tail vein injection only CD57^high^ cells generated liver metastases, without an increase in overall metastatic rate. CD57^high^ U-NB1 also formed more metastases of large size.

### In patient NB of all disease stages strong expression of CD57 is associated with undifferentiated NB cells

Our experimental data were obtained from cell lines of patients with stroma-poor NB. We therefore investigated biopsies of stroma-poor NB. Poorly differentiated and differentiating NB were informative, as they contained both undifferentiated and differentiating tumor cells. In these tumors strong expression of CD57 was preferentially associated with cytologically undifferentiated NB cells across all disease stages. Accordingly, CD57^high^ cells were more often found in undifferentiated and poorly differentiated NB. This is consistent with the notion that CD57-expressing undifferentiated NB cells mirror embryonic or early fetal sympathetic neuroblasts [8]. CD57^high^ cells at diagnosis were not increased in metastasizing disease, in line with a previous study that included ganglioneuromas and ganglioneuroblastomas [16].

### CD57^high^ NB cells after chemotherapy

Following chemotherapy of patients the number of CD57^high^ NB cells within the residual tumor tended to increase. This may be due to selection of CD57^high^ NB cells, assuming that they are less chemosensitive than CD57^low^ cells. Alternatively, CD57 expression may be induced by chemotherapy, a presumption supported by the appearance of CD57^high^ cells in many NB that were devoid of CD57-positive cells prior to chemotherapy.

### Possible mechanisms leading to CD57-associated characteristics of NB cells

CD57 has been shown to mediate invasion and migration of neural crest stem cells [6], [7] and has been associated with metastasis of melanoma, a neural crest stem cell-derived malignancy [17]. As CD57 defines migrating neural crest stem cells, from which NB is thought to arise, it appears plausible that some NB cells have co-opted from NCSC their CD57-associated propensity for migration and invasion. On a more mechanistic level CD57 is known to promote cell-cell interactions [14] and to mediate adherence of cells to laminin of the extracellular matrix via binding to integrins [18], [19]. The latter mechanisms may have contributed to enhanced invasion of CD57^high^ U-NB1 cells.

That adherence of CD57^high^ NB cells to HUVEC and the invasion of CD57^high^ SK-N-BE(2)-C cells into matrix was not inhibited by anti-CD57 blocking antibodies *in vitro* seems to argue against a general role of CD57 in metastasis. However, these *in vitro* finding do not rule out that experimental metastasis *in vivo* could be inhibited by blocking antibodies. Regardless of the exact role of CD57 in experimental metastasis, CD57 expression did not correlate with metastatic disease in patients. Strong expression of CD57 may decrease shedding of NB cells from the primary tumor by increasing homophilic interactions between tumor cells. This may prevent any positive effect of CD57 on late metastasis suggested in the experimental metastasis assay from being operative in patients.

In conclusion, strong expression of CD57 is associated with aggressive attributes of NB cells *ex situ* and with undifferentiated and, to some degree, residual post-chemotherapy NB cells in patients.

## Materials and Methods

### Cell culture

The human neuroblastoma cell line SK-N-BE(2)-C, a *MYCN*-amplified, 1p-deleted cell line established from a bone marrow biopsy of a child with disseminated neuroblastoma after repeated courses of chemotherapy and radiotherapy [13], was obtained from ATCC, Manassas, USA. SK-N-BE(2)-C cells were cultured in a 1∶1 mixture of Eagle's Minimum Essential Medium (LGC Promochem, Teddington, UK) and Ham's F12 (PAA, Pasching, Austria) supplemented with 10% heat-inactivated fetal bovine serum (FBS; Biochrom, Berlin, Germany), 2 mM L-glutamine and penicillin/streptomycin (Invitrogen, Carlsbad, USA) and 0.1 mM nonessential amino acids (Invitrogen) in an atmosphere of 5% CO_2_ at 37°C.

U-NB1 cells were established from a 4 year-old patient with a *MYCN*-non-amplified abdominal neuroblastoma without deletion of 1p36. Dissociated cells were cultured in DMEM/F12, 2% B27 (Invitrogen), 20 ng/ml recombinant human epidermal growth factor (EGF; Strathmann Biotech, Hamburg, Germany), 20 ng/ml recombinant human basic fibroblast growth factor (bFGF; Strathmann Biotech), 20 ng/ml recombinant human leukemia inhibitory factor (LIF; Millipore, Temecula, USA), 10 I.E./ml (5 µg/ml) Heparin (Roche Diagnostics, Mannheim, Germany) and 2 mM L-glutamine and penicillin/streptomycin in a humidified incubator at 37°C in 5% CO_2_. Within 1 week after seeding of the dissociated cells nonadherent spheres were observed. Experiments with U-NB1 cells were performed between passages 1 to 13.

Human umbilical vein endothelial cells (HUVEC; Promocell, Heidelberg, Germany) were cultivated in Endothelial Cell Basal Media (Lonza, Walkersville, MD) supplemented with EGM Single Quots (Lonza), 20 ng/ml recombinant human vascular endothelial growth factor (rhVEGF, R&D Systems, Minneapolis, MN) and 20% FBS (Biochrom) in an atmosphere of 5% CO_2_ at 37°C.

C6 and G14 glioma cells were cultivated in Ham's F12 (PAA) supplemented with 15% horse serum (Biochrom), 2.5% heat -inactivated FBS (Biochrom) and 2 mM L-glutamine at 37°C in an atmosphere of 5% CO_2_.

### FACS cell – sorting

CD57^high^ and CD57^low^ subpopulations of NB cells were isolated using phycoerythrin (PE)–conjugated mouse monoclonal CD57 antibody (Abcam, Cambridge, UK) and the FACSAria™ cell-sorting system (BD Biosciences). 10^7^ cells were incubated at 4°C in PBS (Biochrom) containing 5 µl CD57 antibody or unspecific PE-conjugated IgM isotype control (BD Biosciences). Cells above the 80^th^ percentile of CD57 expression were sorted by flow cytometry into a CD57^high^ fraction, cells below the 20^th^ percentile of CD57 expression into a CD57^low^ fraction.

### Soft agar clonogenicity assay

0.6% soft agar was prepared using low melting point agarose (LMP agarose; Sigma, Taufkirchen, Germany) in Ham's F12 medium (Invitrogen), EMEM (Invitrogen), 10% FBS and additives and added to 24-well plates. SK-N-BE(2)-C cells were separated by flow cytometry into CD57^high^ and CD57^low^ fractions. A single cell suspension of viable cells in 0.3% top agar was added. Plates were incubated at 37°C in growth medium replaced every 3 days for 28 days until colony formation was observed and stained with 5 mg/ml 3–(4, 5–Dimethylthiazol-2–yl)–2, 5–diphenyltetrazoliumbromid (MTT; Sigma, Taufkirchen, Germany) to visualize colonies.

### Sphere - forming assay

U-NB1 and SK-N-BE(2)-C cells growing as spheres were sorted into CD57^high^ and CD57^low^ fractions as described above. Sorted cells were resuspended at a density of 1 viable cell/µl in serum–free DMEM/F12 containing 2% B27, 20 ng/ml rhEGF, 20 ng/ml bFGF, 20 ng/ml LIF, 10 I.E./ml (5 µg/ml) heparin, 2 mM L-glutamine and penicillin/streptomycin. 1 ml of this suspension was added into each well of a 24–well plate with 14 mm cover slips at the bottom of the wells to avoid adherence. For the formation of second–generation spheres CD57^high^ neurospheres were dissociated and either left unfractionated or FACS-sorted into the two CD57 fractions and re–plated at clonal density. Third-generation spheres were generated in the same manner.

### Cytology

To detect peripherin and glial fibrillary acidic protein (GFAP) cells were seeded onto poly–L-lysine coated (SK-N-BE(2)-C) or on collagen type I–coated (U-NB1) glass cover slips and grown overnight. Cells were fixed with 95% ethanol and 5% glacial acetic acid at −20°C, blocked with 0.3 M glycine followed by 10% donkey serum (NDS; Jackson ImmunoResearch, West Grove, USA), 10% goat serum (NGS; Sigma) and 0.5% BSA. Cells were incubated with rabbit anti–peripherin (Invitrogen; 1∶1000) and with anti-GFAP (clone G–A–5; Sigma; 1∶100) in PBS with 0.5% BSA, 1% NDS and 1% NGS for one hour at room temperature. Alexa 488-conjugated donkey anti–rabbit IgG (Invitrogen; 1∶500) and Alexa Fluor 594-conjugated goat anti–mouse IgG_1_ (Invitrogen; 1∶100) were used as secondary antibodies. Nuclei were stained with DAPI. Cells were analyzed using an AX70 PROVIS microscope (Olympus, Hamburg, Germany). For quantification 200 cells per group were analyzed at 400× and 600× magnification and scored as having red, green, red/green or unstained cytoplasm.

For immunocytological detection of CD57, cells were incubated at room temperature in PBS containing 5 µl PE–conjugated mouse monoclonal NK-1 CD57 antibody (Abcam, Cambridge, UK) or unspecific PE-conjugated IgM isotype control (BD Biosciences). Cells were washed, seeded on cover slips in 24-well plates and cultured over night. Subsequently, cells were incubated with HNK-1 anti-CD57 antibody (BD Biosciences) at RT for 1 h, followed by incubation with a secondary anti-mouse IgM-FITC antibody (BD Biosciences). Nuclei were stained with DAPI. Confocal microscopy was performed using the LSM 710 laser scanning microscope (Carl Zeiss, Jena, Germany).

### Forced differentiation assay

NB cells from neurospheres were sorted into CD57^high^ and CD57^low^ fractions, plated either on poly–L-lysine–coated (SK-N-BE(2)-C) or on collagen type I–coated glass cover slips (U-NB1) at a concentration of 10^3^ cells/ml and allowed to adhere overnight in growth media supplemented with 10% FBS. Medium was replaced with media containing 2% FBS and differentiation-inducing cytokines. To differentiate, SK-N-BE(2)-C cells were treated with all–trans retinoic acid (ATRA; Sigma; 20 µM) or with human neuregulin1–β1 (NRG-1-β1; R&D Systems; 5 nM) for 10 days. For differentiation of U-NB1 cells 1 µM ATRA was used for 14 days. Differentiation medium was changed every 3 days. Differentiation was assessed by immunofluorescence cytology. For quantification of differentiation at least 144 colonies per group were analyzed at 400× and 600× magnification and scored as having colonies containing cells with red, green, red and green or unstained cytoplasm.

### Blocking function of the HNK-1 anti-CD57 antibody

C6 glioma cells do not express CD57 because they lack beta-1,3-glucuronyltransferase (GlcAT-P), a rate limiting enzyme of CD57 biosynthesis [20]. In contrast, C6 cells transfected with GlcAT-P cDNA (named G14 cells, [20]) express CD57. To verify the blocking capacity of the HNK-1 anti-CD57 antibody (BD) a 24-well transwell chamber system (8 µm pore size; BD Biosciences) was used. The bottom surface of the insert was coated with 10 µg/ml mouse laminin (Invitrogen) over night at 4°C. C6 and G14 cells were labeled with DiI and incubated in serum-free alpha MEM medium (Gibco) with active or heat-inactivated blocking anti-CD57 antibody for 10 min at 37°C. 1.25×10^5^ cells were seeded in the top chamber. The bottom chamber was filled with serum-free alpha MEM medium. Cells were fixed 24 h post seeding with 3.7% paraformaldehyde. Membranes were cut out from the insert and mounted for detection of cells that migrated through the membrane. 24 low power visual fields were assessed using an inverse microscope (AX70; Olympus, Hamburg, Germany).

### Matrigel invasion assay

BD biocoat tumor invasion system plates (BD Biosciences) containing a matrigel-coated PET membrane with 8 micron pore size were used. NB cells were sorted for the expression of CD57 by flow cytometry and stained with 10 µg/ml 1.1′-dioctadecyl-3,3,3′,3′-tetramethyl-indocarbocyanine perchlorate (DiI, Molecular Probes, Göttingen, Germany). Cells were incubated with 30 µg/µl blocking anti-CD57 antibody (HNK-1, BD Pharmingen) or with heat-inactivated antibody as control for 10 min at 37°C. 2.5×10^4^ cells per well were seeded in RPMI 1640 medium without serum into the upper chamber. The lower chamber was filled with RPMI 1640 containing 10% FCS. Cells were fixed 24 h post seeding with 3.7% paraformaldehyde. Membranes were cut out from the insert and mounted for detection of cells that migrated through the membrane. 24 low power visual fields were assessed using an inverse microscope.

### Adhesion assay on HUVEC

HUVEC were grown to complete monolayers in 24-well plates in Endothelial Cell Basal (EBM) Media (Lonza, Walkersville, MD) supplemented with EGM Single Quots (Lonza), 20 ng/ml recombinant human vascular endothelial growth factor (R&D Systems, Minneapolis, MN) and 20% FBS. NB cells were sorted for the expression of CD57 by flow cytometry and stained with 10 µg/ml 1.1′-dioctadecyl-3,3,3′,3′-tetramethyl-indocarbocyanine perchlorate (DiI, Molecular Probes, Goettingen, Germany). Cells were incubated with 30 µg/µl blocking anti-CD57 antibody (HNK-1, BD Pharmingen) or with heat-inactivated antibody as control for 10 min at 37°C. 2.5×10^4^ NB cells were seeded per well on top of the HUVEC monolayer. After one hour under normal SK-N-BE(2)-C or U-NB1 culture conditions, respectively, cells were washed twice and fixed with 3.7% PFA. DiI-labeled cells per low magnification visual field were determined using an inverted microscope. 24 different visual fields were analyzed.

### Orthotopic NB model

6–8 week old male RAG^−/−^/common γ-chain^−/−^ mice bred in the Animal Research Center of the University of Ulm were anesthetized with ketamin (Pfizer, Berlin, Germany; 25 mg/ml) and rompun (Bayer, Leverkusen, Germany; 20 mg/ml). After laparatomy 1×10^4^ viable U-NB1 cells suspended in 50 µl of a 1∶3 mixture of BD Matrigel™ High Concentration (BD Biosciences) and DMEM/F12 medium without supplements were injected with a 27–gauge needle into the left adrenal gland. Tumors were resected and single cell suspensions were generated by mincing the tumor followed by chemical dissociation with Liberase Blendzyme 1 (Roche). Cells were sorted by FACS into CD57^high^ and CD57^low^ fractions and transplanted again orthotopically as described above. Mice were monitored twice a week for the presence of tumors by palpation and for morbidity.

### Stable transduction of U-NB1 cells with firefly luciferase and detection of luciferase activity

U-NB1 cells were stably transduced with the retroviral expression vector pLib ELN (ProQuinase GmbH, Freiburg, Germany) carrying an enhanced luciferase and neomycin fusion gene and selected for luciferase–expressing neomycin–resistant cells. Luciferase activity was determined using the Luciferase Assay System (Promega Corporation, Madison, USA) and a luminometer (Berthold Technologies, Bad Wildbad, Germany) according to the manufacturers' recommendations.

### Metastasis model

CD57^high^ and CD57^low^ fractions of U-NB1 cells expressing luciferase were generated by FACS sorting. 24 h post sorting 2×10^6^ cells suspended in PBS were injected into the tail vein of 7–10 week old NOD scid gamma (NSG) mice. Bioluminescence indicating tumor growth was monitored every 2 weeks starting 3 weeks after tumor cell injection. Mice were anesthetized with isofluran (Abbott, Wiesbaden-Delkenheim, Germany). Luciferin (Synchem, Felsberg/Altenburg, Germany; 100 mg/kg body weight) was injected intraperitoneally and images were taken using the Xenogen *In vivo* Imaging System IVIS 200 (Caliper Life Sciences, Hopkinton, USA) and Living Images software (Caliper Life Sciences).

### Histology of metastases

Mice were sacrificed 60–80 days after tumor cell injection. Liver, lung, pelvis and tail of all mice and pancreas, fallopian tubes and soft tissue of selected mice were formalin-fixed for 2 h. Pelvises and tails were decalcified in 10% EDTA for 5–7 days. Tissues were embedded in paraffin, serially sectioned, stained with H&E and analysed for the presence of metastases using an AX70 PROVIS microscope (Olympus).

### Immunohistochemistry of patient tumors

Immunohistochemistry was performed on paraffin-embedded tissue sections from the archived tissue files of the Institute of Pathology, University Medical Center Ulm, Germany. Staining was performed according to standard protocols using the EnVision kit (Dako, Carpinteria, CA). As primary CD57 antibody the clone NK1 (IgM) was used (Menrini, Newcastle, UK) at a dilution of 1∶20 after pretreatment of the slides with pronase (1 mg/1 ml). Evaluation was carried out in a semiquantative fashion using CD57-positive natural killer cells that were always present in the tumors as positive internal controls.

### Statistical analysis

Statistical analysis was performed using the PASW statistics software version 18.0 (IBM, Chicago, Illinois).

### Ethics statement

Scientific use of the human tumor tissue employed in this study was approved by the ethics committees of the Ludwig-Maximilians-Universität (permit number 280-07) and of the Klinikum Augsburg (permit of 11.2.2005). Written consent of the patient's parents was obtained.

The animal studies were approved by the Regierungspräsidium Tübingen (permit number 986). All animal experiments were carried out in strict accordance with institutional and state guidelines for animal welfare and all efforts were made to minimize suffering.

## Supporting Information

Figure S1 **HNK-1 anti-CD57 antibody blocks CD57 function.** (**A**) **G14 cells, but not parental C6 glioma cells, express CD57.** Parental C6 glioma cells and G14 cells, i.e. C6 glioma cells overexpressing beta-1,3-glucuronyltransferase, a rate limiting enzyme of CD57 biosynthesis, were assayed for expression of CD57 by immunocytology. Nuclei are stained by DAPI. (**B**) **HNK-1 anti-CD57 antibody blocks migration of C6 glioma cells overexpressing CD57.** C6 and G14 cells were labeled with DiI and incubated with active or heat-inactivated blocking anti-CD57 antibody and added to laminin-coated transwell chambers. 24 hours post seeding cells that had invaded into and migrated through the membrane were fixed. (**C**) **The NK-1 anti-CD57 antibody used for sorting is rapidly internalized and new CD57 epitopes are swiftly presented on the cell surface.** SK-N-BE(2)-C cells were stained with NK-1 anti-CD57-PE (red) used for sorting and plated on cover slips in 24-well plates. 24 h post-staining, cells were incubated with unlabeled HNK-1 anti-CD57 followed by anti-mouse IgM FITC (green) and analyzed by confocal microscopy.
(TIF)

Figure S2 **Histology confirms the presence of metastases detected by bioimaging.** Mice were sacrificed 60–80 days post tumor cell injection. Livers were formalin-fixed, paraffin-embedded and H&E-stained. Tail roots were formalin-fixed, decalcified, paraffin-embedded, H&E-stained and screened for metastases. The histology of mice identified in Fig. 7 is shown.
(TIF)
