# Supplementary figures and images for: CD57high Neuroblastoma Cells Have Aggressive Attributes Ex Situ and an Undifferentiated Phenotype in Patients
Source: PLoS One. 2012 Aug 10;7(8):e42025. doi: 10.1371/journal.pone.0042025 (PMC3416815; doi:10.1371/journal.pone.0042025)

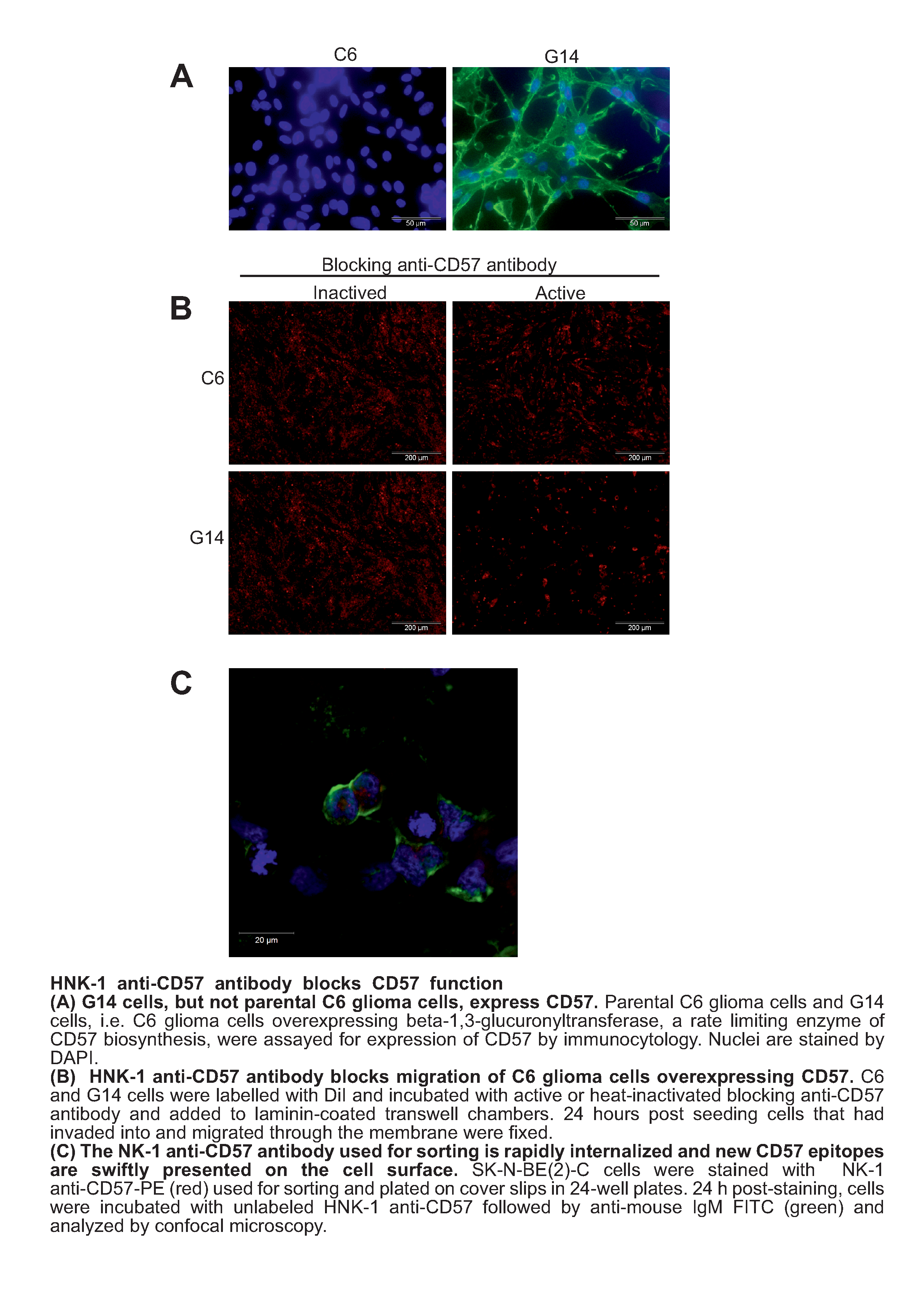

Supplement: Figure S1 — HNK-1 anti-CD57 antibody blocks CD57 function. (A) G14 cells, but not parental C6 glioma cells, express CD57. Parental C6 glioma cells and G14 cells, i.e. C6 glioma cells overexpressing beta-1,3-glucuronyltransferase, a rate limiting enzyme of CD57 biosynthesis, were assayed for expression of CD57 by immunocytology. Nuclei are stained by DAPI. (B) HNK-1 anti-CD57 antibody blocks migration of C6 glioma cells overexpressing CD57. C6 and G14 cells were labeled with DiI and incubated with active or heat-inactivated blocking anti-CD57 antibody and added to laminin-coated transwell chambers. 24 hours post seeding cells that had invaded into and migrated through the membrane were fixed. (C) The NK-1 anti-CD57 antibody used for sorting is rapidly internalized and new CD57 epitopes are swiftly presented on the cell surface. SK-N-BE(2)-C cells were stained with NK-1 anti-CD57-PE (red) used for sorting and plated on cover slips in 24-well plates. 24 h post-staining, cells were incubated with unlabeled HNK-1 anti-CD57 followed by anti-mouse IgM FITC (green) and analyzed by confocal microscopy. (TIF) [file pone.0042025.s001.tif]

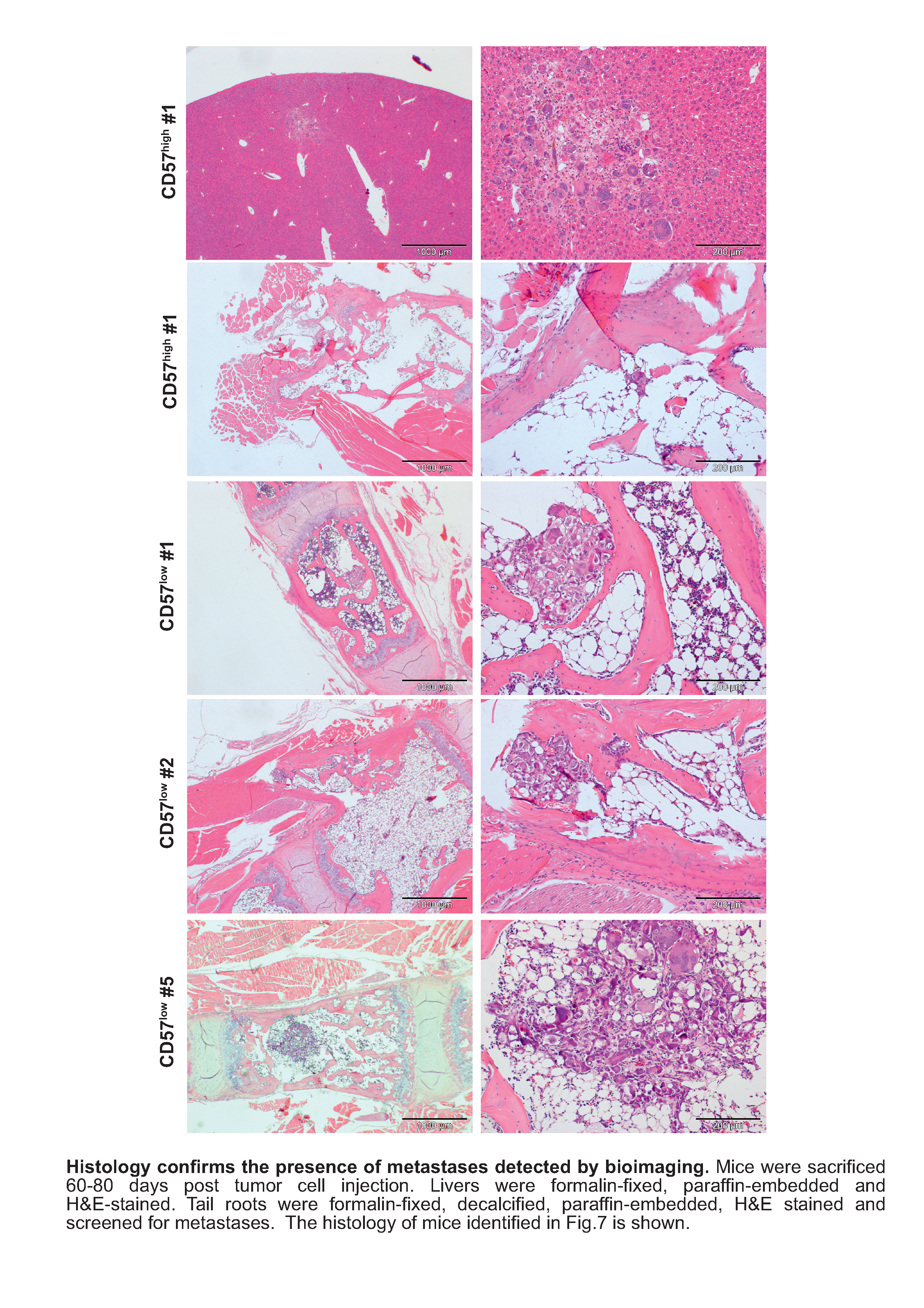

Supplement: Figure S2 — Histology confirms the presence of metastases detected by bioimaging. Mice were sacrificed 60–80 days post tumor cell injection. Livers were formalin-fixed, paraffin-embedded and H&E-stained. Tail roots were formalin-fixed, decalcified, paraffin-embedded, H&E-stained and screened for metastases. The histology of mice identified in Fig. 7 is shown. (TIF) [file pone.0042025.s002.tif]
